# Prognostic Significance of Lung and Cava Vein Ultrasound in Elderly Patients Admitted for Acute Heart Failure: PROFUND-IC Registry Analysis

**DOI:** 10.3390/jcm11154591

**Published:** 2022-08-05

**Authors:** Sara Pérez-Herrero, Noel Lorenzo-Villalba, Elena Urbano, Beatriz Sánchez-Sauce, Fernando Aguilar-Rodríguez, Máximo Bernabeu-Wittel, Rocio Garcia-Alonso, Llanos Soler-Rangel, Francisco Trapiello-Valbuena, Alejandra Garcia-García, Jose Manuel Casas-Rojo, Luis Beltrán-Romero, Lucia De Jorge-Huerta, Juan Igor Molina-Puente, Emmanuel Andrès, Rosario Iguarán-Bermúdez, Manuel Méndez-Bailón

**Affiliations:** 1 Departamento de Medicina, Servicio de Medicina Interna Hospital Clínico San Carlos, Universidad Complutense de Madrid, Instituto de Investigación Sanitaria del Hospital Clinico San Carlos (IdISSC), 28040 Madrid, Spain; 2 Service de Médecine Interne, Diabète et Maladies Métaboliques, Hôpitaux Universitaires de Strasbooug, 67000 Strasbourg, France; 3 Servicio de Medicina Interna, Hospital Fundacion de Alcorcon, 28922 Madrid, Spain; 4 Servicio de Medicina Interna, Hospital 12 de Octubre, 29010 Málaga, Spain; 5 Servicio de Medicina Interna, Hospital Virgen del Rocio, 41013 Sevilla, Spain; 6 Servicio de Medicina Interna, Complejo Asistencial de Ávila, 05004 Ávila, Spain; 7 Servicio de Medicina Interna, Hospital Infanta Sofia, San Sebastian de los Reyes, 28702 Madrid, Spain; 8 Servicio de Medicina Interna, Hospital Central de Asturias, 33011 Oviedo, Spain; 9 Servicio de Medicina Interna, Hospital Gregorio Marañon de Madrid, 28922 Madrid, Spain; 10 Servicio de Medicina Interna, Hospital Infanta Cristina, Parla, 28981 Madrid, Spain

**Keywords:** heart failure, clinical ultrasound, B lines, inferior vena cava, mortality

## Abstract

Introduction: Heart failure is an extremely prevalent disease in the elderly population of the world. Most patients present signs and symptoms of decompensation of the disease due to worsening congestion. This congestion has been clinically assessed through clinical signs and symptoms and complementary imaging tests, such as chest radiography. Recently, pulmonary and inferior vena cava ultrasound has been shown to be useful in assessing congestion but its prognostic significance in elderly patients has been less well evaluated. Objectives: This study aims to compare the clinical and radiological characteristics and predictive values for mortality in patients admitted for heart failure through the determination of B lines by lung ultrasound and the degree of collapsibility of the inferior vena cava (IVC). Secondarily, the study aims to assess the prediction of 30-day mortality based on the diameter of the IVC by means of the ROC curve. Methods: This is an observational cohort study based on data collected in the PROFUND-IC study, a nationwide multicentric registry of patients admitted with decompensated heart failure. Data were collected from these patients between October 2020 and April 2022. Results: A total of 482 patients were entered into the PROFUND-IC registry between October 2020 and April 2022. Bedside clinical ultrasound was performed during admission in 301 patients (64.3%). The number of patients with more than 6 B-lines on lung ultrasound amounted to 194 (66%). Statistically significant differences in 30-day mortality (22.1% vs. 9.2%; *p* = 0.01) were found in these patients. The sum of patients with IVC collapsibility of less than 50% amounted to 195 (67%). Regarding prognostic value, collapsibility data were significant for the number of admissions in the last year (12.5% vs. 5.5%; *p* = 0.04), in-hospital mortality (10.1% vs. 3.3%, *p* = 0.04) and 30-day mortality (22.6% vs. 8.1%; *p* < 0.01), but not for readmissions. Regarding the prognostic value of IVC diameter for 30-day mortality, the area under the ROC curve (AUC) was 0.73, with a *p* < 0.01. The curve cut-off point with the highest sensitivity (70%) and specificity (70.3%) was for an IVC value of 22.5 mm. In the logistic regression analysis, we observed that the variable most associated with patient survival at 30 days was the presence of a collapsible inferior vena cava, with more than 50% OR 0.359 (CI 0.139–0.926; *p* = 0.034). Conclusions: The subgroups of patients analyzed with more than six B lines per field and IVC collapsibility less than or equal to 50%, as measured by clinical ultrasound, had higher 30-day mortality rates than patients who did not fall into these subgroups. IVC diameter may be a good independent predictor of 30-day mortality in patients with decompensated heart failure. Comparing both ultrasound variables, it seems that in our population, the assessment of the inferior vena cava may be more associated with short-term prognosis than the pulmonary congestion variables assessed by B lines.

## 1. Introduction

Heart failure (HF) is a syndrome that affects more than 10% of patients over 80 years of age. Most of these patients have a high burden of comorbid conditions, such as renal failure, diabetes, or chronic obstructive pulmonary disease [1,2,3,4]. During the natural course of HF, patients are frequently admitted for acute clinical decompensation of the disease. This clinical decompensation is usually due to the appearance of symptoms of congestion, leading to edema and increased dyspnea, which contribute to emergency room visits that require intravenous diuretics and hospital admission [1,2].

The assessment of congestion in heart failure is mostly based on clinical evaluation using the Framingham criteria for its diagnosis. However, we know that the clinical reliability of these criteria is lower in elderly patients [5]. Prediction of elevated cardiac filling pressures by single clinical signs of congestion is often unreliable. The emergence of increasingly portable and lightweight ultrasound scanners has made it possible to begin to assess congestion in patients with HF by detecting dilatation and collapsibility of the inferior vena cava and the presence of B lines [6].

Pulmonary congestion (PC) is one of the major characteristics of HF [6,7,8]. The importance of PC in the disease course of HF has been confirmed by numerous clinical trials, and the presence of PC is shown to be associated with a significantly increased risk of mortality and rehospitalization in HF patients [6,9]. Multiple studies have demonstrated the usefulness of assessment of pulmonary congestion from a prognostic point of view. However, techniques have not been compared with each other, nor have they been extensively evaluated in the population of elderly patients admitted to internal medicine or geriatric services [6].

The aim of the study was to compare the clinical characteristics of patients admitted for acute heart failure, according to the number of Kerley B lines at admission (>6 or equal to or below (6)), to compare the clinical characteristics of these patients by determining the collapsibility of the inferior vein cava (IVC), to predict the probability of hospital readmission and early mortality at 30 days from follow-up, and to evaluate the predictive value of 30-day mortality based on IVC diameter measured by clinical ultrasound.

## 2. Methods

### 2.1. Study Design

This was a prospective observational cohort study that included 482 patients admitted for acute heart failure as the primary diagnosis. Data were collected from the PROFUND-IC registry in the period October 2020–April 2022. The PROFUND-IC registry is a nationwide multicenter registry of patients, with heart failure and comorbid conditions defined as the presence of two or more chronic diseases. The inclusion criteria include diagnosis of heart failure as the main diagnosis upon admission, NT-PROBNP values of >1500 pg/mL, and written consent to enter the study.

### 2.2. Variables

Baseline epidemiological, clinical, radiological, and therapeutic variables were analyzed, as well as mortality and early readmission at 30 days. Data were collected retrospectively through an on-line data capture system. HF classification was based on left ventricular ejection fraction or LVEF (reduced EF: <40%, intermediate EF: 40–49%, preserved EF: ≥50%) according to the European guidelines. The New York Heart Association (NYHA) classification was used to assess the functional category of heart failure at baseline and at least two weeks prior to hospital admission (class I: no limitation of physical activity; class II: slight limitation of physical activity and comfortable at rest; class III: marked limitation of physical activity and comfortable at rest; class IV: inability to carry out any physical activity without discomfort and symptoms of heart failure or angina syndrome present even at rest). Comorbid conditions, such as arterial hypertension, diabetes mellitus, dyslipidemia, chronic obstructive pulmonary disease, and atrial fibrillation diagnosed before or during hospital admission, were collected. We also considered the previous history of SARS-CoV-2 virus infection.

Laboratory variables were collected upon admission and included complete blood count, electrolytes, renal function tests, blood gases, and coagulation tests, as well as NT-PROBNP and carbohydrate antigen 125 (CA 125) values. Chest radiography and ultrasound during the first 72 h of hospitalization were also collected.

Information on the treatment received for heart failure included the use of angiotensin converting enzyme inhibitors, angiotensin II receptor antagonists, sacubitril/valsartan, beta-blockers, anti-aldosterone agents, and diuretics. The maximum dose of furosemide received during admission was also recorded.

Multiorgan ultrasound was performed during the first 72 h of hospital admission and data regarding the number of B lines, as well as the maximum vena cava diameter and its collapsibility (more and less than 50%), were collected. The determination of B lines was performed following a protocol of eight lung ultrasound areas, as recommended in the clinical ultrasound consensus documents, and the determination of IVC diameter and collapsibility was also performed following these recommendations. The ultrasound was performed by an external physician who received training in clinical ultrasound. This ultrasound was performed by the investigators who participated in the registry and who had received at least one month of training in clinical ultrasound. The ultrasound was carried out by multiple investigators. The ultrasound equipment used was an Esaote MyLab™25.

Finally, these variables were evaluated with respect to overall readmission and 30-day mortality.

### 2.3. Statistical Analysis

A descriptive analysis of the clinical characteristics of the patients was performed. We also conducted a bivariate analysis comparing the clinical characteristics of the patients with respect to the collapsibility of the vena cava (greater or less than 50%) and the presence of six or more B lines determined. Student’s *t*-test was used for quantitative variables and chi-square test for qualitative tests, and *p* < 0.05 was considered statistically significant. A ROC curve analysis was performed to evaluate the variable IVC diameter (mm) with respect to cumulative mortality during the follow-ups. For this purpose, the area under the ROC curve was measured with the statistical significance set below *p* < 0.05. We performed a multivariate logistic regression analysis including prognostic variables of heart failure, such as age, left ventricular ejection fraction and ultrasound variables, such as B lines and collapsibility of the inferior vena cava. SPSS 21.0 software was used for statistical analysis.

### 2.4. Ethical Aspects

All participants signed the informed consent. This study was conducted according to the Declaration of Helsinki, the Belmont Report, and other related documents. Data confidentiality was maintained in accordance with the Data Protection Act. This study was approved by the Ethics Committee del Hospital Fundacion Alcorcon de Madrid.

## 3. Results

A total of 482 patients were included in the PROFUND-IC registry during the period of October 2020 to April 2022. Multiorgan clinical ultrasound was performed in 301 patients (64.3%). Table 1 shows the clinical, analytical, and epidemiological characteristics of patients according to the number of B lines. Table 2 shows these data regarding the inferior cava vein collapsibility (≥50% vs. <50%).

A total of 194 (66%) patients presented with more than six B lines on the clinical ultrasound. Women were found to have more B lines than men (60.3% vs. 39.7%), respectively. Patients with more than six B lines also presented greater values of NT-proBNP upon admission (10,096.98 vs. 7405.7 pg/mL; *p* < 0.05). Interestingly, patients with previous SARS infection were found to have less B lines than those without this antecedent (9.8% vs. 20.2%; *p* = 0.01).

Vena cava collapsibility below 50% was noted in 195 (67%) patients. There were no significant statistical differences regarding the epidemiological characteristics or treatment between both groups, except for the presence of obstructive sleep apnea and higher CA-125 value in patients with less collapsibility (17.4% vs. 4.2%, *p* = 0.01; 104.27 vs. 69.31, *p* < 0.05, respectively). Patients with lower vena cava collapsibility presented higher rates of pleural effusion on the chest ray and lung ultrasound (75.7% vs. 44.1%; *p* < 0.01 y 63.1% vs. 35.1%; *p* < 0.01, respectively) and a higher number of Kerley B lines on the chest rays (51.3% vs. 29.7%; *p* < 0.01). We also observed that higher cava vein diameter was related to lower collapsibility (21.69 vs. 19.14, *p* < 0.01). With respect to the prognostic value of collapsibility, we found significant results concerning in-hospital mortality (10.1% vs. 3.3%, *p* = 0.04) and 30-day mortality (22.6% vs. 8.1%; *p* < 0.01), but not for readmissions. In relation to the results analyzed between the doses of furosemide prescribed at the time of discharge in subjects with greater than six B lines and patients with six lines or less, we did not obtain statistically significant differences (66.12 ± 37.16 mgr vs. 66.43 ± 42.10 mgr with *p* = 0.95) between the two groups. We did not observe differences between greater or less than 50% collapsibility of the inferior vena cava (61.71 ± 38.51 mgr vs. 68.70 ± 39.34 mgrp = 0.18). In relation to valve replacement, cardiac catheterization and use of dialysis, there were no statistical differences between the groups classified by pulmonary ultrasound and inferior vena cava.

**Figure 1:**
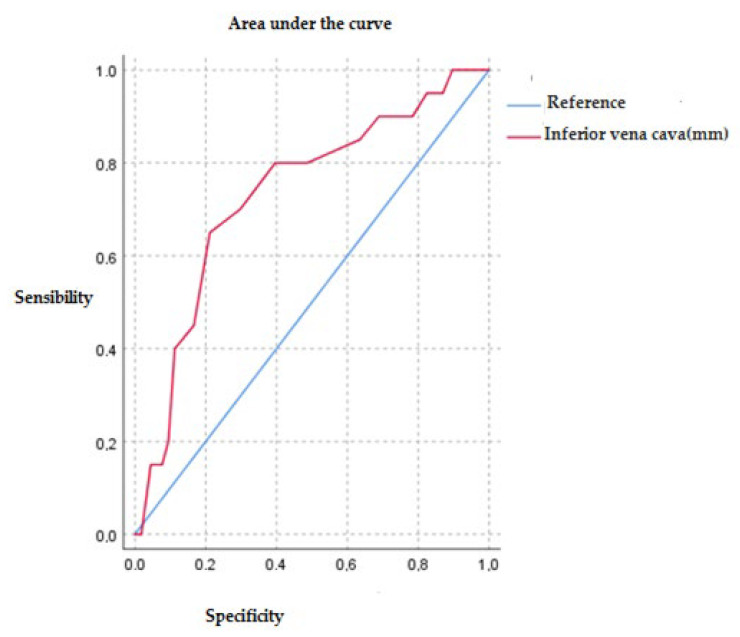
Analysis of the area under the curve for inferior vena cava diameter (mm) and 30-day mortality.

**Figure 2:**
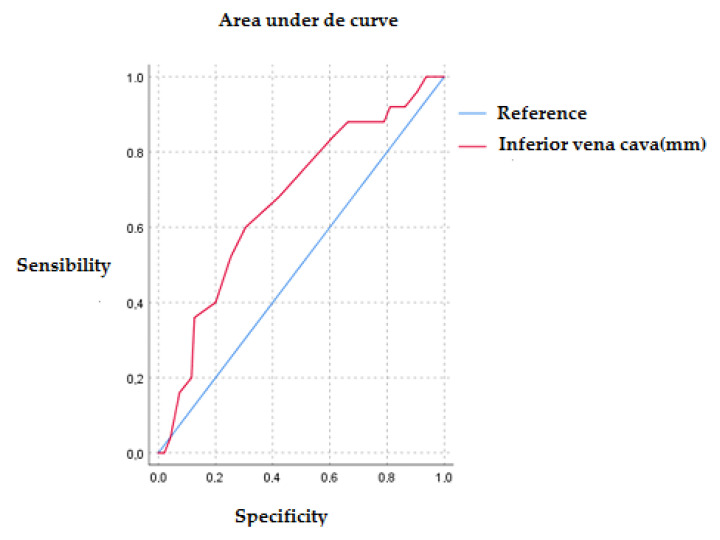
ROC curve of IVC diameter and 30-day mortality for patients with more than 6 B lines on lung ultrasound.

Figure 1 shows the area under the curve for IVC values regarding 30-day mortality at discharge. In relation to the prognostic value of IVC diameter and 30-day mortality, we observed that the area under the curve was 0.73, *p* < 0.01. The curve cut-off point with the highest sensitivity (70%) and specificity (70.3%) was for an IVC value of 22.5 mm. We performed an ROC curve analysis with the subgroup of patients who presented more than six B-lines by lung ultrasound. The area under the curve obtained was 0.67 with a *p* = 0.004 for 30-day mortality, with IVC diameter in millimeters. The cut-off point obtained was 22.5 mm, with a sensitivity of 60% and a specificity of 70%. The 1S plot of the ROC curve can be found in Figure 2.

Table 3 shows the results of the logistic regression analysis, in which it can be observed that collapsibility equal to and greater than 50% was associated with better prognosis in the series analyzed (OR 0.359 CI 0.139–0.926 *p* = 0.034).

## 4. Discussion

Our study shows that collapsibility of the inferior vena cava equal to or greater than 50%, as assessed by clinical ultrasound, in elderly patients admitted for HF is associated with a lower cumulative mortality rate at the 30-day follow-up. In addition, the presence of a 22.5 cm vena cava was associated with a specificity and sensitivity of 70% for mortality at that follow-up time. These findings are congruent with those observed in other studies, showing that elevations in IVC diameter are associated with higher right atrial pressure and a greater presence of pulmonary hypertension [10,11,12]. These factors have been shown to be predictors of poor prognosis in the short and medium term and can be reliably assessed by clinical ultrasound.

In addition, the absence of collapsibility of the inferior vena cava was associated with a greater presence of congestion in the lungs and with an increase in Kerley B lines and pleural effusion. Another parameter currently used to determine the degree of congestion in heart failure is the CA-125 level. In this study, CA-125 levels were significantly associated with less IVC collapsibility and were, therefore, more predictive of the degree of congestion [13,14,15,16]. This association between decreased inferior vena cava collapsibility values and elevated CA-125 values suggests that these parameters are more elevated in those subjects with a higher degree of pulmonary hypertension and right HF.

Most of the patients in this cohort (66%) had a higher number of B lines in the first 72 h after hospitalization. In other words, the patients presented a high proportion of pulmonary congestion attributable to HF, due to left ventricular dysfunction. The high number of B lines was also associated with a higher rate of death at 30 days. These findings have been observed in other lung ultrasound studies performed in younger patients, such as the study by Platz et al. [17], in which patients with more than seven B lines had a higher rate of death and readmission for HF during follow-up. Considering these preliminary findings, we have divided our sample into patients with more than six B lines or six B lines or less.

The role of moderate/severe cognitive impairment in this pathology is of interest. In this respect, we assume that patients with greater cognitive impairment were not correctly evaluated, due to the difficulties derived from appropriate anamnesis including cooperation and more complex physical examination [18]. This may suggest that the use of diuretics in this type of patient has not been fully intensified during their stay at the emergency room and hospitalization. This would result in a greater level of pulmonary congestion with more B lines compared to other patients.

Regarding the different imaging techniques performed in these patients with respect to clinical ultrasound, the value of the chest X-ray was comparable to that of the ultrasound. More B lines and the less collapsibility of the IVC was associated with greater presence of pleural effusion and Kerley B lines on the chest X-ray.

The use of IVC ultrasound is increasing in patients with heart failure. When the degree of congestion in the patient increases, there is a dilation of the diameter of the IVC and less collapsibility during inspiration. This is relevant in patients with obstructive sleep apnea syndrome who presented an absence of IVC collapsibility. Due to these repeated micro-apneas during sleep, this subgroup of patients usually has a higher risk of pulmonary hypertension, and a higher risk of right ventricular dysfunction or chronic respiratory failure, also known as Cor pulmonale [19]. In patients with OSA and other obesity-related conditions, it is more common to find a dilated vena cava, with no collapsibility, and other cardiovascular risk factors [19].

Regarding mortality in patients with heart failure, the main predictors known to date are age, depression, NYHA III/IV class, and renal failure [20]. This study found a higher percentage of death in the group of patients with more than six B lines on the lung ultrasound than in the group with no IVC collapsibility. In relation to the diameter of the IVC, the area under the curve was 0.73 (*p* < 0.01), making it reasonable to suggest that the diameter of 22.5 mm of the IVC has an acceptable ability to predict the mortality in these patients. However, the current sample is not very large and does not reach a statistically significant result.

There is no consensus regarding the use of clinical ultrasound for the evaluation of patients with heart failure, in comparison with the usual methods of examination. Clinical ultrasound could be added to the arsenal of variables already known as an additional tool to predict mortality in these patients. The most relevant parameter is the collapsibility of the IVC and its diameter, since there are studies that show that a larger diameter of the IVC implies greater mortality, more readmissions, and worse renal function in these patients [21,22]. These results, together with those of our study, reaffirm the clinical relevance of an ultrasound assessment of the IVC as additional information in patients with HF. It is also a safe technique, fast, and easily reproducible technique that can improve traditional clinical evaluation. The main limitation is that in more than 20% of patients with HF, it is often difficult to assess the vena cava due to air interposition, obesity, and poor ultrasound window.

The present study has the following limitations: (1) loss of patients due to difficulty in performing the technique, lack of equipment availability or patients with acute decompensated heart failure who do not meet the criteria to enter the PROFUND-IC study, (2) difference in the training in clinical ultrasound of the different clinical, which can enhance the differences between the ultrasound values studied and decrease their accuracy. In this sense, we must state that most of the researchers of the registry were trained in clinical ultrasound, which makes the multicenter results very reproducible. (3) Finally, there was a lack of follow-up of part of the population studied, thus losing part of the prognostic information.

## 5. Conclusions

The subgroup of patients with more than six B lines on the ultrasound of the lungs in HF presented higher mortality rates at 30 days compared to patients with less than six B lines. The subgroup of patients with IVC collapsibility less than or equal to 50% had higher mortality rates at 30 days compared to patients with collapsibility of >50%. The IVC diameter may be a good independent predictor of 30-day mortality in patients with decompensated heart failure. Comparing both ultrasound variables, it seems that in our population, the assessment of the inferior vena cava may be more associated with short-term prognosis than the pulmonary congestion variables assessed by B lines.

## Figures and Tables

**Table 1:** Baseline characteristics of patients with heart failure according to the presence of B lines (>6 or ≤6).

Variable	B Lines > 6	B Lines ≤ 6	*p*
	*n* = 194	*N* = 100	
COMORBIDITIES			
Age, years (median, SD)	82.54 (σ = 10.52)	83.86 (σ = 8.41)	n.s.
Sex			
Men (N,%)	77 (39.7%)	41 (41.0%)	*p* < 0.05
Woman (N,%)	117 (60.3%)	59 (59.0%)	*p* < 0.05
Hypertension (N,%)	173 (89.6%)	93 (93.0%)	n.s.
Diabetes mellitus (N,%)	92 (47.4%)	44 (44.0%)	n.s.
Dyslipidemia (N,%)	133 (68.6%)	62 (62.0%)	n.s.
Coronary heart disease (N,%)	65 (65.7%)	34 (34.3%)	n.s
Valvular heart disease (N,%)	38 (19.7%)	32 (32.7)	*p* < 0.05.
COPD (N,%)	41 (21.1%)	19 (19.0%)	n.s.
Mild-severe cognitive impairment (N,%)	21 (10.8%)	4 (4.0%)	*p* < 0.05
OSA (N,%)	29 (14.9%)	11 (11.0%)	n.s.
Tobacco			
Previous tobacco (N,%)	46 (23.8%)	35 (35.0%)	n.s.
Active (N,%)	13 (6.7%)	4 (4.0%)	n.s.
CRF (GRF < 60 mL/min) (N,%)	90 (46.4%)	48 (48%)	n.s.
AF (N,%)	134 (69.1%)	66 (66.0%)	n.s.
Moderate/severe aortic stenosis (N,%)	32 (16.7%)	22 (22.0%)	n.s.
Moderate/severe mitral insufficiency (N,%)	53 (27.5%)	34 (34.3%)	n.s.
Previous COVID-19 infection (N,%)	19 (9.8%)	20 (20.2%)	0.01
Clinical, analytical, and radiological characteristics			
Systolic blood pressure (mmHg) (media, SD)	138.22 (σ = 27.01)	139.28 (σ = 25.02)	n.s.
Everest score (media, SD)	8.32 ± 3.06	6.82 ± 2.67	*p* < 0.05
LVEF (media, SD)	49.59 (σ = 13.20)	54.10 (σ = 11.53)	*p* < 0.05
<40%	50 (26.5%)	14 (21.9%)	
41–49%	25 (13.2%)	8 (8.1%)	
>50%	114 (60.3%)	77 (77.8%)	
NYHA III/IV (N,%)	67 (34.7%)	36 (36%)	n.s.
NT-proBNP (pg/mL) (media, SD)	10,096.98	7405.7	*p* < 0.05
Oxygen saturation (media, SD)	92.62 (σ = 4.37)	94.12 (σ = 4.42)	*p* < 0.05
Dyspnea (N,%)	188 (96.9%)	93 (94.0%)	n.s.
Cardiomegaly on chest rays (N,%)	173 (90.1%)	86 (86.0%)	n.s.
Pleural effusion on chest rays (N,%)	126 (65.6%)	46 (46.0%)	*p* < 0.01
Pleural effusion on echography (N,%)	125 (64.8%)	33 (33.0%)	*p* < 0.01
Kerley lines on chest rays (N,%)	102 (53.1%)	28 (28.6%)	*p* < 0.01
IVC diameter (mm) (media, SD)	20.78 (σ = 5.65)	21.02 (σ = 8.62)	n.s.
Hemoglobin g/dl (media, SD)	11.74 (σ = 1.94)	11.71 (σ = 1.88)	n.s.
Creatinine mg/dl (media, SD)	1.34 (σ = 0.72)	1.32 (σ = 0.63)	n.s.
CA-125 (media, SD)	94.62 (σ = 103.49)	89.08 (σ = 117.58)	n.s.
Treatment			
ACE inhibitors (N,%)	54 (28.6%)	22 (22.4%)	n.s.
Angiotensin II receptor antagonists (N,%)	42 (22.3%)	20 (20.4%)	n.s.
Sacubitril-valsartan (N,%)	18 (9.6%)	7 (7.1%)	n.s.
Beta-blockers (N,%)	120 (63.5%)	59 (60.2%)	n.s.
SGLT2 (N,%)	39 (20.9%)	16 (16.7%)	n.s.
Maximum dose of furosemide received, mg during 24 h (media, SD)	124.21 (σ = 112.37)	86.84 (σ = 65.67)	*p* < 0.05
Non-invasive mechanical ventilation (NIMV)	12(6.3%)	1 (1%)	
Variables predicting mortality			
4 or more admissions in the last year (N,%)	24 (12.6%)	8 (8.0%)	n.s.
In-hospital mortality (N,%)	17 (9.0%)	5 (5.2%)	n.s.
30-day mortality (N,%)	33 (22.1%)	7 (9.2%)	*p* = 0.01
Readmission (N,%)	34 (23.3%)	14 (19.2%)	n.s.

**Legend. COPD:** chronic obstructive pulmonary disease, **OSA**: obstructive sleep apnea; **CRF**: chronic renal failure; **GFR**: glomerular filtration rate; **AF**: atrial fibrillation; **LVEF**: left ventricular ejection fraction; **IVC**: inferior vena cava; **ACE:** angiotensin converting enzyme inhibitors; **SGLT2:** sodium-glucose cotransporter-2 (SGLT2) inhibitors.

**Table 2:** Baseline characteristics of patients with heart failure according to inferior vena cava collapsibility (>50% or ≤50%).

Variables	Inferior Vena Cava Collapsibility ≤ 50%)	Inferior Cava Vein Collapsibility > 50%	*p*
	*n* = 195	*n* = 95	
COMORBIDITIES			
Age, years (median, SD)	83.04 (σ = 9.77)	82.70 (σ = 10.24)	n.s.
Sex			
Men (N,%)	78 (40.0%)	39 (41.1%)	n.s.
Women (N,%)	117 (60.0%)	56 (58.9%)	n.s.
Hypertension (N,%)	175 (90.2%)	88 (92.6%)	n.s.
Diabetes mellitus (N,%)	87 (44.6%)	48 (50.5%)	n.s.
Dyslipidemia (N,%)	127 (65.1%)	64 (64.0%)	n.s
Coronary heart disease (N,%)	61 (31.3%)	36 (37.9%)	n.s
Valvular heart disease (N,%)	29 (19.7%)	29 (31.2%)	*p* < 0.05
COPD (N,%)	41 (21.0%)	17 (17.9%)	n.s.
Mild-severe cognitive impairment (N,%)	17 (8.7%)	8 (8.4%)	n.s.
OSA (N,%)	34 (17.4%)	4 (4.2%)	*p* = 0.01
Tobacco			
Previous tobacco (N,%)	55 (28.4%)	26 (27.4)	n.s.
Active (N,%)	10 (5.2%)	6 (6.3%)	n.s.
CRF (GRF < 60 mL/min) (media, SD)	95 (48.7%)	40 (42.1%)	n.s.
AF (N,%)	131 (67.2%)	65 (68.4%)	n.s.
Moderate/severe aortic stenosis (N,%)	33 (17.2%)	18 (35.3%)	n.s.
Moderate/severe mitral insufficiency (N,%)	59 (30.6%)	28 (29.5%)	n.s.
Previous COVID 19 infection (N,%)	22 (11.3%)	15(16.0%)	n.s.
Clinical, analytical, and radiological characteristics			
Systolic Blood pressure (mmHg) (media, SD)	138.26 (σ = 26.64)	139.82 (σ = 25.97)	n.s.
Everest Score (media, SD)	8.50 ± 2.91	6.49 ± 2.72	*p* < 0.05
LVEF (media, SD)	50.55 (σ = 13.21)	51.91 (σ = 12.23)	n.s.
<40%	46 (24.2%)	19 (20%)	
41–49%	24 (12.6%)	9 (9.5%)	
>50%	120 (63.2%)	67 (70.5%)	
NYHA III/IV (N,%)	67 (34.6%)	35 (36.8%)	n.s.
NT-proBNP pg/mL(media, SD)	9939.15	7816.31	n.s.
Oxygen saturation (media, SD)	92.90 (σ = 4.36)	93.37 (σ = 4.71)	n.s.
Dyspnea (N,%)	187 (93.5%)	91 (95.8%)	n.s.
Cardiomegaly on chest ray (N,%)	179 (91.8%)	76 (81.7%)	*p* < 0.05
Pleural effusion on chest ray (N,%)	128 (75.7%)	41 (44.1%)	*p* < 0.01
Pleural effusion on clinical ultrasound (N,%)	123 (63.1%)	33 (35.1%)	*p* < 0.01
Kerley lines on chest ray (N,%)	100 (51.3%)	27 (29.7%)	*p* < 0.01
IVC diameter (mm) (media, SD)	21.69 (σ = 5.48)	19.14 (σ = 8.7)	*p* < 0.01
Hemoglobin g/dl (media, SD)	11.73 (σ = 1.99)	11.81 (σ = 1.74)	n.s.
Creatinine mg/dl (media, SD)	1.37 (σ = 0.71)	1.27 (σ = 0.65)	n.s.
CA 125 (media, SD)	104.27 (σ = 111.71)	69.31 (σ = 99.26)	*p* < 0.05
Treatment			
ACE inhibitors (N,%)	49 (25.9%)	25 (26.6%)	n.s.
The maximum dose of furosemide received mg (media, SD)	118.00 (σ = 104.8)	98.39 (σ = 90.88)	n.s.
Non invasive mechanical ventilation (NIMV) (N,%)	11 (5.7%)	1 (1.1%)	n.s.
Angiotensin II receptor antagonists (N,%)	43 (22.8%)	18 (19.1%)	n.s.
Sacubitril- valsartan (N,%)	15 (7.9%)	11 (11.7%)	n.s.
Beta-blockers (N,%)	117 (65.7%)	61 (64.9%)	n.s.
SGLT2I (N,%)	39 (20.9%)	17 (18.3%)	n.s.
Variables predicting mortality			
Four or more admissions in the last year (N,%)	24 (12.5%)	5 (5.5%)	0.04
In-hospital mortality (N,%)	19 (10.1%)	3 (3.3%)	0.04
30-day mortality (N,%)	33 (22.6%)	6 (8.1%)	*p* < 0.01
Readmission (N,%)	28 (19.9%)	18 (24.7%)	n.s.

**Legend. COPD:** chronic obstructive pulmonary disease, OSA: obstructive sleep apnea; **CRF**: chronic renal failure; **GFR**: glomerular filtration rate; **AF**: atrial fibrillation; **LVEF**: left ventricular ejection fraction; **IVC**: inferior vena cava; **ACE:** angiotensin converting enzyme inhibitors; **SGLT2:** sodium-glucose cotransporter-2 (SGLT2) inhibitors.

**Table 3:** Logistic regression analysis of clinical and ultrasound variables with 30-day cumulative mortality.

Variable	Odds Ratio	Confidence Interval	*p*
Age	1.003	0.966–1.041	0.881
Left ventricular ejection fraction	0.992	0.964–1.021	0.565
>6 B lines	2.019	0.803–5.076	0.135
Inferior vena cava collapsibility (≥50%)	0.359	0.139–0.926	0.034

## Data Availability

Data are contained within the article.
